# Bibliometric and visual analysis of global publications on kaempferol

**DOI:** 10.3389/fnut.2024.1442574

**Published:** 2024-08-16

**Authors:** Ruying Tang, Longfei Lin, Yuling Liu, Hui Li

**Affiliations:** ^1^Institute of Chinese Materia Medica, China Academy of Chinese Medical Sciences, Beijing, China; ^2^Institute of Traditional Chinese Medicine Health Industry, China Academy of Chinese Medical Sciences, Nanchang, China

**Keywords:** kaempferol, bibliometrics, CiteSpace, VOSviewer, Scimago Graphica, Web of Science, pharmacology

## Abstract

**Introduction:**

Kaempferol, a flavonoid found in numerous foods and medicinal plants, offers a range of health benefits such as anti-inflammatory, antioxidant, antiviral, anticancer, cardioprotective, and neuroprotective effects.

**Methods:**

Herein, a bibliometric and visual analysis of global publications on kaempferol was performed to map the evolution of frontiers and hotspots in the field. Using the search string TS = kaempferol, bibliometric data for this analysis was extracted from the Web of Science Core Collection database and analyzed using the VOSviewer, CiteSpace, and Scimago Graphica software.

**Results:**

As a result, by February 26, 2024, 11,214 publications were identified, comprising articles (*n* = 10,746, 96%) and review articles (*n* = 468, 4%). Globally, the annual number of kaempferol publications surpassed 100 per year since 2000, exceeded 500 per year since 2018, and further crossed the threshold of 1,000 per year starting in 2022. The major contributing countries were China, the United States of America, and India, while the top three institutes of the citations of kaempferol were the Chinese Academy of Sciences, Consejo Superio de Investigaciones Cientficas, and Uniersidade do Porto. These publications were mainly published in agricultural and food chemistry journals, food chemistry, and phytochemistry.

**Discussion:**

The keywords frequently mentioned include phenolic compounds, antioxidant activity, flavonoids, NF-kappa B, inflammation, bioactive compounds, etc. Anti-inflammation, anti-oxidation, and anti-cancer have consistently been the focus of kaempferol research, while cardiovascular protection, neuroprotection, antiviral, and anti-bacterial effects have emerged as recent highlights. The field of kaempferol research is thriving.

## 1 Introduction

Generally, the daily intake of natural plant substances has been highlighted by scholarly research for their numerous health benefits and their potential bioactivity in preventing various diseases (1–4). Among these natural plant substances, flavonoids have been isolated from plants and proven to have multiple preventive and therapeutic effects on diseases (5, 6). Especially, kaempferol, a bioactive flavonoid monomer extracted from different plants, has garnered extensive global research and scholarly attention due to its properties, including anti-inflammatory, anti-tumor, anti-oxidative stress anti-allergy, anti-viral, and cardiac protection effects (7–12). Kaempferol stands as a flavonoid that has been isolated and purified through modern scientific methods. It is widely found in medicinal plants such as Galangal, Rhizoma Drynariae, Semen Cuscutae, *Ginkgo Biloba*, Leaf of Eucommla Ulmoides, etc. (8, 13). Kaempferol is a tetrahydroxy flavone that presents as yellow needle-shaped crystals with four hydroxy groups positioned at the 3, 5, 7, and 4′ positions (14). Researchers have been summarizing the presence of kaempferol in various parts of plants, including seeds, leaves, fruits, and flowers. Besides, kaempferol is also widely distributed in natural sources of medical plants. It can be prepared and purified using microporous adsorption resin column chromatography, silica gel column chromatography, and other chromatographic techniques (15). Currently, kaempferol represents more than 5.7 billion US dollars in the global consumer market of medicine and food (16). As a medicine and health compound, kaempferol has broad worldwide research and development potential.

Kaempferol has the molecular formula C_15_H_10_O_6_, with a relative molecular weight 286.23. Its chemical name is 3,5,7,4′-tetrahydroxyflavone. It is slightly soluble in water but in ether, alkali, and hot ethanol (8, 17). Like most flavonoids, kaempferol often exists in various natural plants, fruits, and vegetables in different glycosides. An aglycone produced by hydrolysis of an acidic substance can improve the total extraction rate of kaempferol (13). In addition, kaempferol, in the form of glycosides, can directly enter the bloodstream and exhibit various biological activities within the human body (18). The antioxidant stress activity of kaempferol has been confirmed. Kaempferol can promote the expression of heme oxygenase-1, inhibit the production of nitric oxide and inducible nitric oxide synthase, and reduce the damage of lipopolysaccharide on RAW264.7 macrophages (8, 19, 20). Furthermore, kaempferol plays a neuroprotective role by inhibiting oxidative stress. Previous studies have also revealed that kaempferol has notable anti-inflammatory effects, including regulating the expression of inflammation-related genes and the activity of pro-inflammatory enzymes and inhibiting the expression of matrix metalloproteinases, adhesion molecules, and transcription factors (8, 21). Besides, considerable studies have shown kaempferol’s efficacy in accelerating cancer cell apoptosis. For instance, *in vitro* experiments have indicated that kaempferol can inhibit the proliferation of malignant tumor cells such as breast, colon, lung, bladder, and liver (9, 22). In addition, studies have demonstrated that kaempferol can increase the number of endothelial progenitor cells in the lumen after treatment with advanced glycosylation end-products in diabetic patients who have suffered a myocardial infarction. This finding implies that kaempferol can improve microvascular complications associated with diabetes (23–25). This kind of valuable research into the therapeutic potential for myocardial protection also bolsters kaempferol’s clinical medicinal research value (26). Meanwhile, kaempferol also exerts potential therapeutic effects in treating cataracts, coughs, fertility inhibition, anti-ulcer, anti-epilepsy, etc. (27, 28). However, a holistic analysis of the scientific literature on kaempferol is missing. Thus, performing a thorough analysis and offering a guide to current research trends is essential for informing ongoing scholarly work.

Bibliometrics focuses on the literature system and the characteristics of bibliometric studies as its subject matter. It employs mathematical and statistical measurement methods to primarily investigate the structure, quantitative relationships, change patterns, and quantitative management of the distribution of literature and information. Furthermore, it delves into the structures, characteristics, and laws of science and technology (29, 30). Bibliometrics mainly takes literature as the research object and analyzes the quantitative attributes of existing literature. The CiteSpace software, developed by Dr. Chen Chaomei and his team, serves as a citation visualization analysis software that has been gradually developed against the background of bibliometrics and data visualization. It is currently the most widely used software in bibliometrics (31, 32). The CiteSpace or VOSviewer software can extract and analyze the semantic contents of the titles, abstracts, and keywords of publications. It performs well in relating them to the citation count data and generating a bubble map to visualize the results (33, 34). These bibliometrics and visualization results assist researchers in grasping the research’s frontier and hotspot. Besides, Scimago Graphica software can aesthetically visualize the results of bibliometric analyzes conducted with tools like CiteSpace or VOSviewer (35). Furthermore, Scimago Graphica software can generate a national geographic map where each node signifies a distinct country. The node’s size is directly proportional to the volume of literature published by that country (36). The connection between nodes represents the cooperation between nations, while the connection’s thickness indicates the cooperation’s closeness (37). Besides, in the co-occurrence analysis graph of VOSviewer software, node and font size represent the frequency of occurrence, the lines between nodes indicate cooperation, and the thickness indicates closeness (38). In addition, in the co-occurrence and cluster analysis results of CiteSpace software, different colors indicate different years. In the spectrum of the cluster analysis graph, the value of the cluster module (Q value) and the mean value of the cluster profile (S value) can be observed (39). The former represents the association of the cluster nodes, and the latter represents the association of the cluster topics. In the outburst map, one should look for the bright red areas, which signify the time range of the outburst (40). Briefly, bibliometrics presents the knowledge structure and frontier trends of a research field utilizing modern techniques such as CiteSpace, VOSviewer, Scimago Graphica, etc., to visualize countries, institutions, authors, journals, documents, and keywords intuitively. To this end, a bibliometric and visual analysis was performed regarding global publications on kaempferol in the Web of Science (WoS) Core Collection database. The aim was to provide total-scale analysis results, track research hotspots, and offer insights for future kaempferol research.

## 2 Materials and methods

### 2.1 Data collection

Literature on kaempferol was retrieved from the Web of Science (WoS) Core Collection database, focusing on journal articles. The MeSH^1^ confirmed the term “kaempferol” with no synonyms found (41). Subsequently, the search term and strategy used for the WoS database was “TS = kaempferol.” Articles from the databases’ inception until February 26, 2024, were searched. The search was limited to English-language publications and included articles and review document types. The data search and export were finalized on February 26, 2024, to prevent any data deviation that subsequent database updates might cause. Ultimately, 11,214 documents were obtained, and the data results were exported in the “txt” format, including “Full Record and Cited References” for subsequent analysis.

### 2.2 Data selection

The inclusion criteria for literature were as follows: (1) the research content of the literature revolved around kaempferol; (2) the literature involved kaempferol-related pharmaceutical experiments, animal experiments, cell experiments, clinical trials, and other studies; and (3) both research papers and review papers could be included. Otherwise, the exclusion criteria for literature were as follows: (1) literature with incomplete information such as title, keywords, journal, and author; (2) literature of news reports, scientific and technological achievements, conference notices, conference papers, call for papers, patents, and other types; and (3) only one duplicate publication was retained.

All 11,214 documents were imported into Endnote X9 literature management software, and two researchers screened them independently to remove duplicate ones and retain their uniqueness. Then, the data was extracted and cross-checked. Any discrepancies were discussed among the other research members to decide on inclusion. In the end, it was confirmed that there were no duplicate documents among the 11,214 documents.

### 2.3 Rationale of the bibliometric tools

The CiteSpace software, developed using Java, is an information visualization tool that employs co-citation analysis theory and the pathFinder algorithm to assess literature in specific fields (42). Consequently, it effectively reveals the research field’s hotspots, development trends, and novel advancements. Besides, VOSviewer is grounded in the principles and methodologies of bibliometrics, employing techniques such as data mining, information processing, and knowledge measurement to represent intricate literature as knowledge maps visually. It uses processes including standardization of similarity, clustering networks based on keywords, and visual representation to generate visual keyword clusters, density maps, and multi-window time-series graphs of keywords, thereby unveiling the latest trends and advancements in a research domain (43). Furthermore, Scimago Graphica is a drag-and-drop data visualization software that simplifies the process of data analysis and chart creation. Users do not need to write complex codes or formulas. They can quickly import data into the software by dragging, dropping, and selecting the appropriate chart type and settings to generate visual charts (44).

### 2.4 Data analysis

Microsoft Excel was used to compile statistics and create visualizations of the annual and cumulative publications (44). Subsequently, the VOSviewer 1.6.20 software facilitated a bibliometric analysis encompassing countries, institutions, authors, co-cited authors, journals, and references per established research methodologies. The method parameters of the VOSviewer were set as Linlog/modularity, and the weight parameter was set according to the number of documents (37, 45). Following that, the analysis results from VOSviewer were exported and saved in the format of “gml.” for the network visualization of Scimago Graphica.

Scimago Graphica 1.0.30 Software was used to display the analysis results from VOSviewer visually (46). In Scimago Graphica’s network visualizations, nodes symbolize entities such as countries/regions, institutes, authors, co-cited authors, journals, and references. Node size corresponds to publication volume; lines denote cooperative ties, line thickness reflects the strength of collaboration, and node colors differentiate clusters (47, 48).

The CiteSpace 6.2 R6 software was utilized to conduct a co-occurrence analysis of the keywords from global publications on kaempferol. The parameters of CiteSpace were set as follows: method: LLR; time slicing: from January 1975 to February 2024; years per slice: 5; term source: all selection; and node type: one at a time (41). Besides, the silhouette value represents the cluster network homogeneity, with a silhouette value greater than 0.7 indicating the high reliability of the results (41, 49). Furthermore, the Q value of the cluster network signifies its modularity, with a Q value greater than 0.5 indicating a significant cluster structure of the network (39, 41). Finally, the timeline plot and the top keywords with the most robust citation bursts of kaempferol’s global publications were visually displayed and exported using CiteSpace.

## 3 Results

### 3.1 Annual publication statistics and analysis results

**Figure 1:**
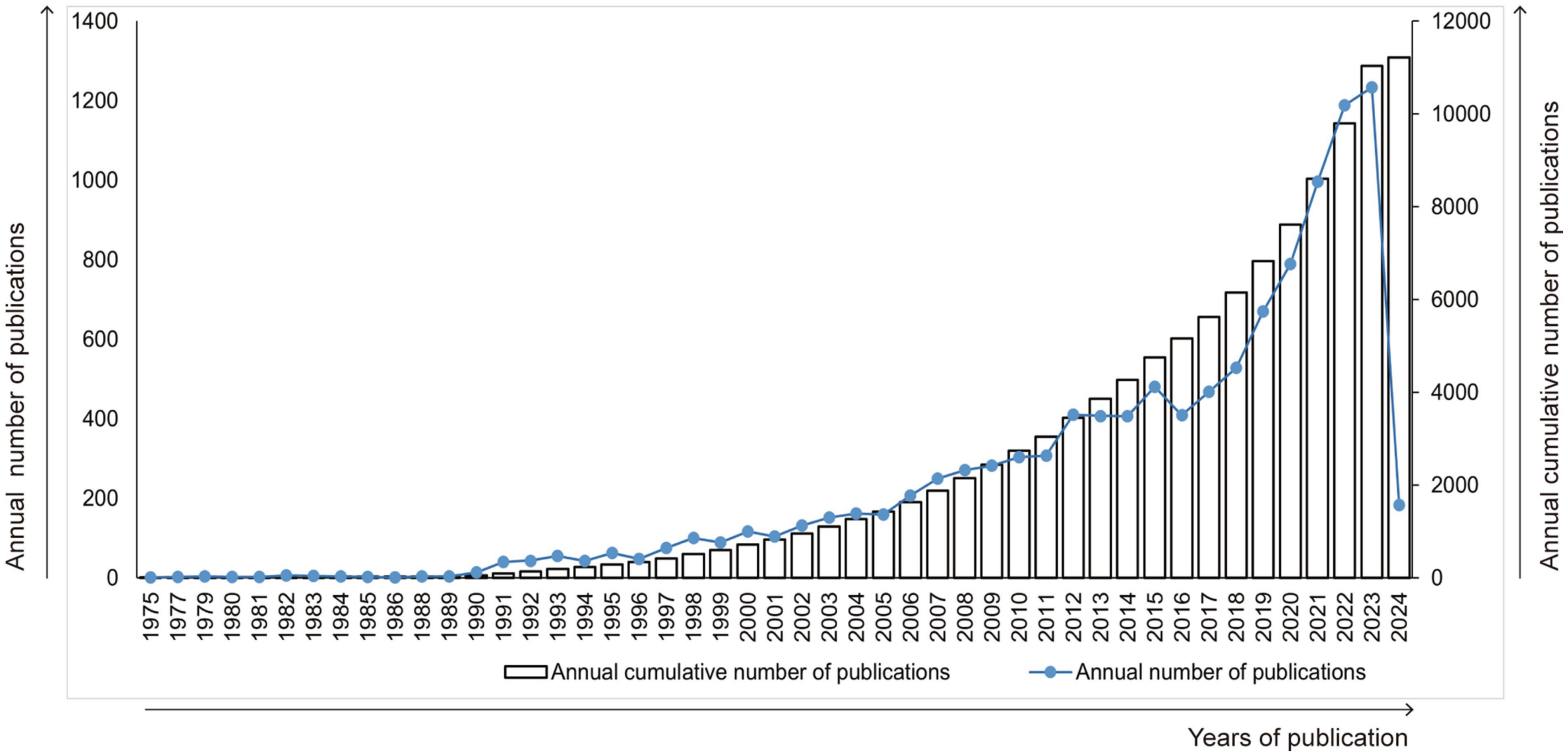
Trend graph of the growth of annual and cumulative annual number of kaempferol’s publications.

As shown in Figure 1, since 1975, research results of kaempferol have been published worldwide. The publication count experienced a gradual increase from 1990 to 2015, but it surged dramatically from 2016 to 2023, peaking with the highest number of publications in 2023 (*n* = 1,234). Besides, since the retrieval time of this study concluded on February 26, 2024, all relevant literature in 2024 could not be included, resulting in a decline in the number of papers published in 2024.

### 3.2 Contribution of countries/regions

**Figure 2:**
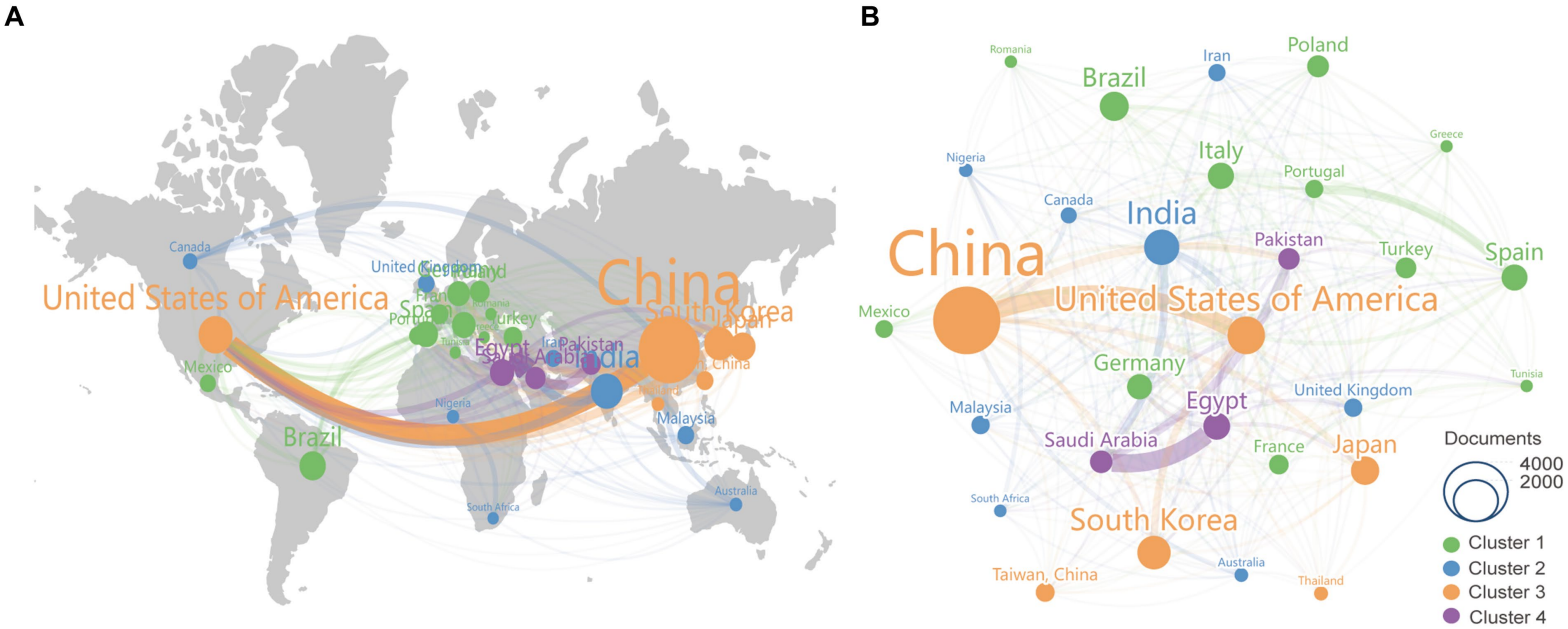
The most productive countries/regions of publications on kaempferol. **(A)** Spatial distribution of kaempferol’s global publications’ top 30 countries/regions. **(B)** Visual display of the top 30 countries/areas of kaempferol’s global publications. VOSviewer was used for analysis; the method was ling/modularity, weight documented, the minimum number of documents of a country was set as 100, and results were visually displayed by Scimago Graphics software. The thickness of the lines indicates the strength of the relationship. The different colors of the circle represent the various clusters of countries/regions. The larger the circle and label, the more kaempferol’s global publications the country has published.

A total of 147 countries/regions participated in the research on kaempferol. Figure 2A presents the spatial distribution of the top 30 countries/regions, while Figure 2B shows a visualization analysis of 30 countries/regions with the publication volume exceeding 100 documents. The size of the nodes corresponds to the document counts, the lines suggest the connections between countries/regions, and the thickness of the lines indicates the strength of the connection between countries/regions. The countries/regions were divided into four clusters based on the degree of cooperation represented by different colors. As clearly shown in Figure 2, China had the most published documents and the closest collaboration with the United States on the research of kaempferol. Besides, Table 1 exhibits detailed information on the document counts, citation counts, total link strength, and link counts of the top 30 countries/regions. According to the results in Table 1, China (*n* = 2,917), the United States of America (*n* = 947), and India (*n* = 832) were the three leading contributors to global kaempferol research publications, while the United States of America (*n* = 60,179), China (*n* = 53,774), and Spain (*n* = 21,402) were the top three countries/regions contributing the most citations of kaempferol publications. The connections between countries/regions were mainly focused on the cooperation between the United States of America (the total link strength was 596) and other countries, including China (the total link strength was 508), Egypt (the total link strength was 410), and India (the total link strength was 301).

**Table 1:** Top 10 the most productive countries/regions of kaempferol’s global publications.

Rank	Country/region	Document	Citation	Total link strength	Link
1	China	2,917	53,774	508	26
2	United States of America	947	60,179	596	29
3	India	832	17,346	301	28
4	South Korea	749	21,374	172	21
5	Brazil	567	12,903	131	21
6	Japan	537	20,837	195	24
7	Egypt	480	7,676	410	29
8	Italy	459	18,716	254	26
9	Spain	453	21,402	258	25
10	Germany	421	18,786	223	27

### 3.3 Contribution of institutes, co-cited journals, authors, and co-cited authors

#### 3.3.1 Contribution of institutes

**Figure 3:**
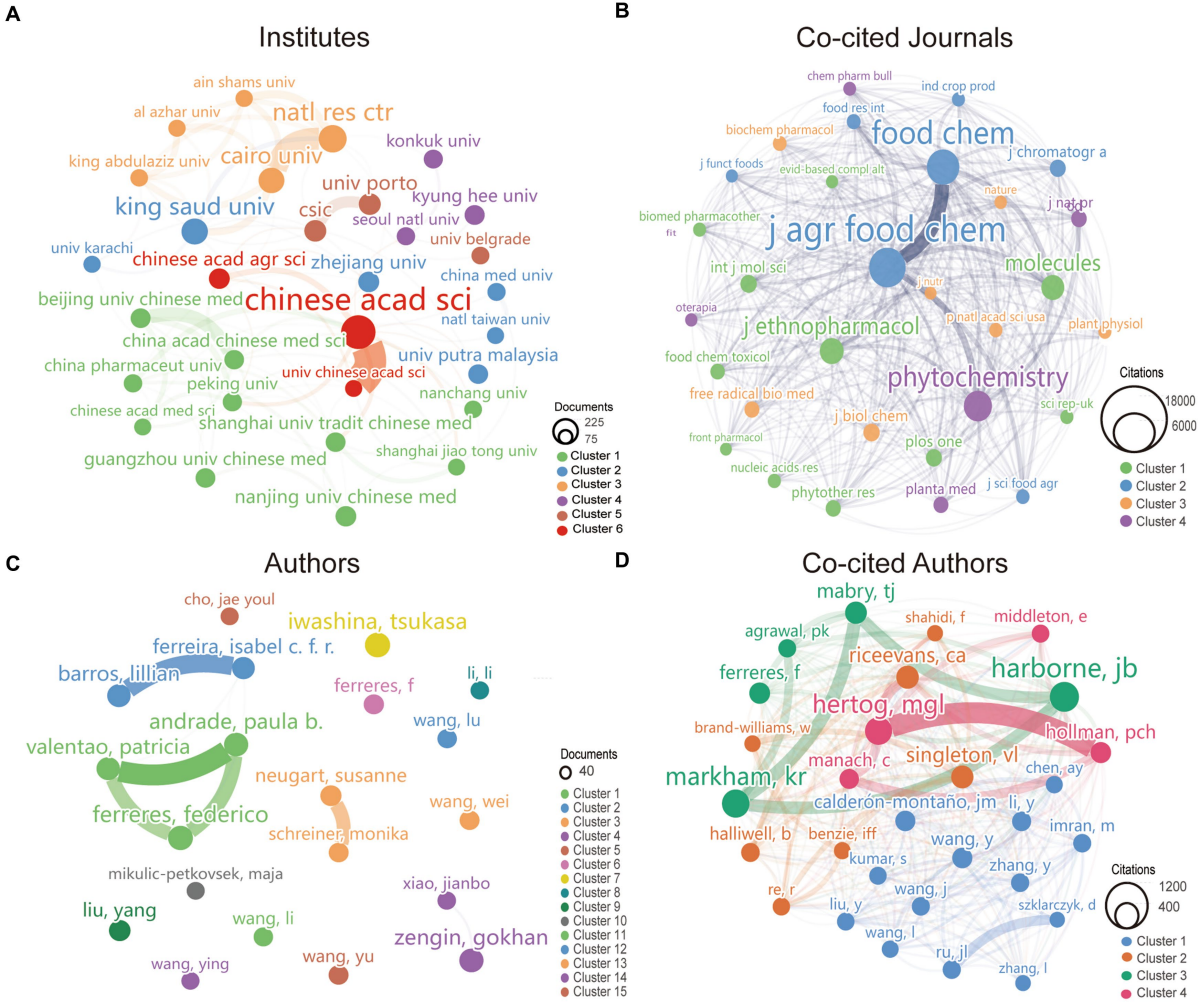
The most productive institutes, co-cited journals, authors, and co-cited authors of publications on kaempferol. **(A)** Visual display of the top 31 institutes of kaempferol’s global publications. The minimum number of documents required by an institute was set at 50. **(B)** Visual display of the top 26 co-cited journals of kaempferol’s global publications. The minimum number of citations of a co-cited journal was set as 2000. **(C)** Visual display of the top 20 authors of kaempferol’s global publications. The minimum number of documents an author has was set at 19. **(D)** Visual display of the top 29 co-cited authors of kaempferol’s global publications. The minimum number of citations for a co-cited author was set at 260. VOSviewer was used for analysis; the method was ling/modularity, weight was documented, and results were displayed visually by Scimago Graphica software. The thickness of the lines indicates the strength of the relationship. The different colors of the circle represented the various clusters of institutes, co-cited journals, authors, or co-cited authors. The larger the circle and label, the more kaempferol’s global publications the institutes, co-cited journals, authors, or authors have published.

A total of 698 institutes participated in the research of kaempferol. According to VOSviewer analysis results, there were 31 institutions with more than 50 global publications related to kaempferol, and the visual network diagram is shown in Figure 3A (the minimum number of documents of an organization was 50). The size of the nodes corresponds to the document counts; the lines suggest the connections between institutes, and the thickness of the lines indicates the strength of the connection between institutes. The institutes were divided into six clusters based on the degree of cooperation represented by different colors. As shown in Figure 3A, the Chinese Academy of Sciences had the most published documents and the closest collaboration with the Chinese Academy of Agricultural Sciences on the research of kaempferol. Table 2 presents detailed information on the top 10 most productive institutes for global publications on kaempferol. According to the results, the Chinese Academy of Sciences (*n* = 214), National Research Centre NRC (*n* = 141), and King Saud University (*n* = 126) ranked as the top three institutes contributing to the global body of kaempferol publications. Besides, the top three institutes in terms of citation counts for international publications on kaempferol were: the Chinese Academy of Sciences (*n* = 5,243), Consejo Superio de Investigaciones Cientficas (*n* = 5,118), and Uniersidade do Porto (*n* = 3,455). The top three in total link strength, indicating prominent collaborative ties, were primarily centered around the Chinese Academy of Sciences (the total link strength was 89), Cairo University (the total link strength was 74), and the National Research Centre NRC (the total link strength was 69).

**Table 2:** Top 10 the most productive institutes of kaempferol’s global publications.

Rank	Institute	County/region	Document	Citation	Total link strength	Link
1	Chinese Academy of Sciences (Chinese acad sci)	China	214	5,243	89	12
2	National Research Centre NRC (natl res ctr)	Egypt	141	2,157	69	8
3	King Saud University (King Saud univ)	Saudi Arabia	126	1945	31	9
4	Cairo University (Cairo univ)	Egypt	121	2,229	74	7
5	Consejo Superio de Investigaciones Cientficas (csic)	Spain	87	5,118	20	4
6	Universidade do Porto (univ Porto)	Portugal	87	3,455	19	3
7	Universidade de São Paulo (univ são paulo)	Brazil	85	2,542	0	0
8	Zhejiang University (zhejiang univ)	China	79	2017	8	8
9	Chinese Academy of Agricultural Sciences (Chinese acad agr sci)	China	78	1,614	19	7
10	Nanjing University of Chinese Medicine (Nanjing University Chinese med)	China	78	1,145	11	5

#### 3.3.2 Distribution of co-cited journals

A total of 1,621 co-cited journals were found in the research on kaempferol. According to VOSviewer analysis results, there were 26 co-cited journals with more than 2,000 global publications on kaempferol, and the visual network diagram is shown in Figure 3B (the minimum number of citations of a co-cited journal was 2,000). The co-cited journals were divided into four clusters based on the degree of cooperation represented by different colors. A co-citation relationship was identified between journals if the same document cited two or more journals. The size of the nodes represents the total frequency of co-citation. A larger node indicates a higher frequency of co-citations, which implies a more significant influence of the journal within the research field of kaempferol. Therefore, as shown in Figure 3B, *the Journal of Agricultural and Food Chemistry had the most citations, indicating that more researchers would publish their studies results of kaempferol in* this journal. Table 3 presents detailed information on the top 10 most cited journals in the global publications on kaempferol. According to the results, *the Journal of Agricultural and Food Chemistry* (*n* = 17,827), *Food Chemistry* (*n* = 14,179), and *Phytochemistry* (*n* = 10,635) were identified as the top three most cited journals in the global publications on kaempferol.

**Table 3:** Top 10 the most productive co-cited journals of kaempferol’s global publications.

Rank	Co-cited journal	Citation	Impact factor	The year of the impact factor
1	Journal of Agricultural and Food Chemistry (j agr food chem)	17,827	5.7	2023
2	Food Chemistry (food chem)	14,179	8.5	2023
3	Phytochemistry (phytochemistry)	10,635	3.2	2023
4	Journal of Ethnopharmacology (j ethnopharmacol)	7,660	4.8	2023
5	Molecules (molecules)	7,091	4.2	2023
6	International Journal of Molecular Sciences (int j mol sci)	3,602	4.9	2023
7	Journal of Biological Chemistry (j biol chem)	3,553	4.0	2023
8	Plos One (plos one)	3,430	2.9	2023
9	Journal of Chromatography A (j chromatogram a)	3,394	3.8	2023
10	Journal of Natural Products (j nat prod)	3,223	3.3	2023

#### 3.3.3 Contribution of authors and co-cited authors

A total of 741 authors performed research on kaempferol. According to VOSviewer analysis results, there were 20 authors with more than 19 global publications on kaempferol, and the visual network diagram is shown in Figure 3C (the minimum number of documents of an author was 19). The size of the nodes corresponds to the document counts; the lines suggest the connections between authors, and the thickness of the lines indicates the strength of the connection between authors. The authors were divided into 15 clusters based on the degree of cooperation represented by different colors. As shown in Figure 3C, Iwashina and Tsukasa from Japan had the most published documents on kaempferol research. Clusters signified close collaboration and guided researchers in identifying potential research partners. In kaempferol research, the closest cooperative relationships were observed between Andrade, Paula Branquinho, Valentao, Patricia, and Ferreres, Federico. Besides, there was also a notable collaboration between Barros, Lillian, Ferreira, Isabel C. F. R., Neugart, Susanne, and Schreiner, Monika.

A total of 47,853 co-cited authors performed research on kaempferol. Among them, 29 co-cited authors with more than 260 citations were selected for visualization analysis, as shown in Figure 3D. A co-citation relationship existed between authors if the same document cited two or more authors. The size of the node represents the total frequency of co-citation. A larger node indicates a higher frequency of co-citations, which implies a more significant influence of the author within the research field of kaempferol. As noted in the analysis results, Harborne JB from the United Kingdom was the most cited author, making significant contributions to and having a far-reaching influence on kaempferol research. Moreover, Harborne JB, Markham KR, and Mabry TJ had a close cooperative relationship.

Table 4 presents detailed information on the top 10 most productive authors and most cited authors of kaempferol publications. According to the results, the top three authors in terms of publications on kaempferol globally were Iwashina, Tsukasa (*n* = 37), National Museum of Nature and Science, Japan; Ferreres, Federico (*n* = 36), CSIC – Centro de Edafologia y Biologia Aplicada del Segura, Spain; and Andrade, Paula Branquinho (*n* = 34), Universidade do Porto, Portugal. Besides, the top three co-cited authors in the global citations of kaempferol publications were Harborne JB (*n* = 939), University of Reading, United Kingdom; Markham KR (*n* = 833), Callaghan Innovation DSIR CHEM DIV, New Zealand, and Rice-Evans CA (*n* = 557), King’s College London, United Kingdom.

**Table 4:** Top 10 the most productive authors and co-cited authors of kaempferol’s global publications.

Rank	Author	Document	County/region	Institute	Co-cited author	Citation	County/region	Institute
1	Iwashina, Tsukasa	37	Japan	National Museum of Nature and Science	Harborne JB	939	United Kingdom	University of Reading
2	Ferreres, Federico	36	Spain	CSIC – Centro de Edafologia y Biologia Aplicada del Segura (CEBAS)	Markham KR	833	New Zealand	Callaghan Innovation DSIR CHEM DIV
3	Andrade, Paula Branquinho	34	Portugal	Universidade do Porto	Rice-Evans CA	557	United Kingdom	King’s College London
4	Zengin, Gokhan	34	Italy	Selcuk University	Mabry TJ	524	United States of America	University of Texas Austin
5	Barros, Lillian	32	Portugal	Instituto Politécnico de Bragança	Ferreres, Federico	475	Spain	CSIC – Centro de Edafologia y Biologia Aplicada del Segura (CEBAS)
6	Valentao, Patricia	32	Portugal	Universidade do Porto	Hollman, PCH	464	Netherlands	Wageningen University
7	Ferreira, Isabel C. F. R.	30	Portugal	Instituto Politécnico de Bragança	Yuzhen Wang	443	China	Inner Mongolia Agricultural University
8	Liu, Yang	27	China	Nanjing Forestry University	J M Calderón-Montaño	440	Spain	University of Seville
9	Neugart, Susanne	26	Germany	Georg-August University Göttingen	Huiyi Li	399	China	Guangdong Pharmaceutical University
10	Schreiner, Monika	23	Germany	Leibniz Institut fur Gemuse- und Zierpflanzenbau (IGZ)	Yin Zhang	362	China	General Hospital of Chinese PLA

### 3.4 Highly co-cited references

Co-cited references were used to study the internal connections between literature and depict scientific development’s dynamic structure. The results revealed that 1,621 co-cited references were closely related to the research topic of kaempferol. According to the citation analysis of documents, which reflected the citations of documents, the top 10 high citations are listed in Table 5. The work titled “A review on the dietary flavonoid kaempferol” by J M Calderón-Montaño had the most citations (439), followed by that titled “TCMSP: a database of systems pharmacology for drug discovery from herbal medicines” by Jinlong Ru (*n* = 364); “Antioxidant activity applying an improved ABTS radical cation decolorization assay” by R Re (*n* = 333); “A review of the dietary flavonoid, kaempferol on human health and cancer chemoprevention” by Allen Y Chen (*n* = 315), and “Structure-antioxidant activity relationships of flavonoids and phenolic acids” by C A Rice-Evans (*n* = 310).

**Table 5:** Top 10 the most productive co-cited references of kaempferol’s global publications.

Rank	Title	First author	Year	Journal	Impact factor	The year of the impact factor	Citation
1	A review of the dietary flavonoid kaempferol	J M Calderón-Montaño	2011	MINI-REVIEWS IN MEDICINAL CHEMISTRY	3.3	2023	439
2	TCMSP: a database of systems pharmacology for drug discovery from herbal medicines	Jinlong Ru	2014	JOURNAL OF CHEMINFORMATICS	7.1	2023	364
3	Antioxidant activity applying an improved ABTS radical cation decolorization assay	R Re	1999	FREE RADICAL BIOLOGY AND MEDICINE	7.1	2023	333
4	A review of the dietary flavonoid kaempferol on human health and cancer chemoprevention	Allen Y Chen	2013	FOOD CHEMISTRY	8.5	2023	315
5	Structure-antioxidant activity relationships of flavonoids and phenolic acids	C A Rice-Evans	1996	FREE RADICAL BIOLOGY AND MEDICINE	7.1	2023	310
6	The ferric reducing ability of plasma (FRAP) as a measure of “antioxidant power”: the FRAP assay	I F Benzie	1996	ANALYTICAL BIOCHEMISTRY	2.6	2023	255
7	C-13 NMR-Studies of Flavonoids 0.3. Naturally Occurring Flavonoid Glycosides and Their Acylated Derivatives	Markham, KR	1978	TETRAHEDRON	2.1	2023	209
8	The effects of plant flavonoids on mammalian cells: implications for inflammation, heart disease, and cancer	E Middleton Jr	2000	PHARMACOLOGICAL REVIEWS	19.3	2023	206
9	Kaempferol and inflammation: From chemistry to medicine	Kasi Pandima Devi	2015	PHARMACOLOGICAL RESEARCH	9.1	2023	201
10	Flavonoids as antioxidants	P G Pietta	2000	JOURNAL OF THE AMERICAN CHEMICAL SOCIETY	14.4	2023	190

### 3.5 Research hotspots analysis results

**Figure 4:**
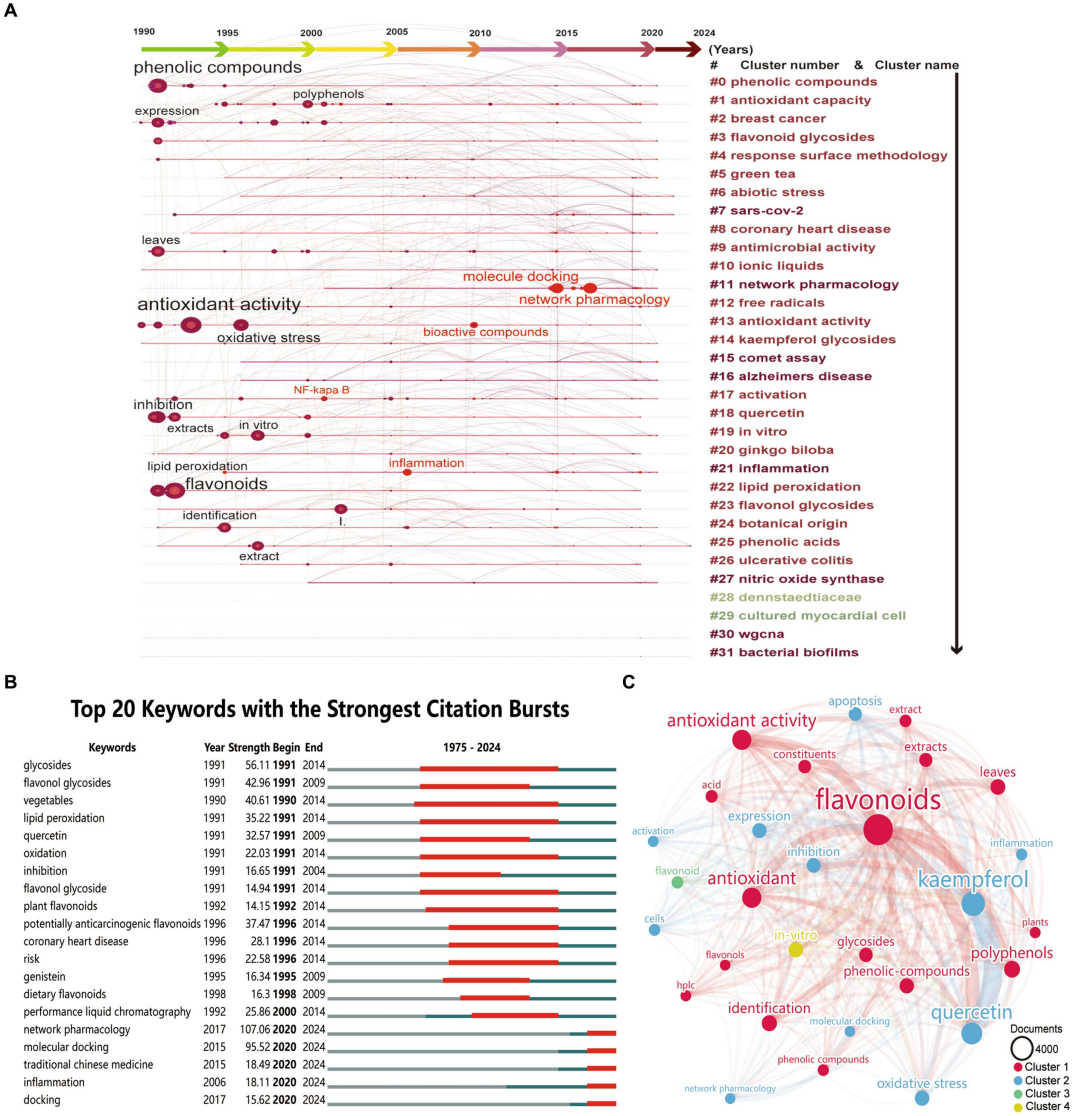
Co-occurrence analysis of keywords of kaempferol’s global publications. **(A)** Timeline plot of keywords co-occurrence clustering of kaempferol’s global publications by CiteSpace. The graph shows the time order from left to right, the results of keyword clustering from top to bottom, and the circles represent keywords. The larger the circle, the more times the keyword appears, and the bright red circle indicates a sudden increase in the keywords. **(B)** Top 20 keywords with the strongest citation bursts of kaempferol’s global publications by CiteSpace. ɣ: 1.0; minimum duration: 2. **(C)** Visual display of the top 30 keywords of kaempferol’s global publications. VOSviewer was used for analysis, the method was linlog/modularity, weight was documents, minimum number of occurrences of a keyword was set as 380, and visual display by Scimago Graphica software. The thickness of the lines indicates the strength of the relationship. The different colors of circle represented the difference clusters of keywords. The larger the circle and label, the more kaempferol’s global publications the keywords have occurred.

A co-occurrence analysis of keywords from global publications on kaempferol was conducted using CiteSpace Software. Keywords encapsulate the core topics of a publication and are instrumental for identifying research hotspots and directions in the field of kaempferol. Herein, the analysis yielded a total of 1,080 distinct keywords in the field of kaempferol research. Figure 4A presents a timeline plot of keyword co-occurrence clustering for global publications on kaempferol, as generated by CiteSpace. The results indicated that research hotspots and directions of kaempferol were mainly focused on “phenolic compounds,” “antioxidant activity,” “flavonoids,” etc. Besides, the keywords that suddenly increased included “NF-kappa B,” “inflammation,” “bioactive compounds,” “molecule docking,” “network pharmacology,” etc. Furthermore, CiteSpace was used to find clusters, and there were 31 clusters in total in the analysis results. The clusters were mainly identified in the research field related to keywords including “phenolic compounds,” “antioxidants capacity,” “breast cancer,” “flavonoid glycosides,” “response surface methodology,” “green tea,” “abiotic stress,” “sars-cov-2,” “coronary heart disease,” “antimicrobial activity,” “ionic liquids,” “network pharmacology,” “free radicals,” “antioxidant activity,” “kaempferol glycosides,” “comet assay,” “Alzheimer’s disease,” “activation,” “quercetin,” “*in vitro*,” “*ginkgo biloba*,” “inflammation,” “lipid peroxidation,” “flavonol glycosides,” “botanical origin,” “phenolic acids,” “ulcerative colitis,” “nitric oxide synthase,” “Dennstaedtiaceae,” “cultured myocardial cell,” “wgcna,” and “bacterial biofilms.” This cluster information provided potential directions and hotspots for further study regarding kaempferol.

Figure 4B highlights the top 20 keywords with the most significant citation bursts(a great change of publications in a short period), with “network pharmacology” and “molecule docking” leading from 2020 to 2024 at 107.06 and 95.52, respectively. This indicated a growing scholarly focus on utilizing network pharmacology and molecular docking techniques to study kaempferol. Besides, the strongest citation bursts were observed in the following order: “glycosides,” 56.11 (from 1991 to 2014), “flavonol glycosides,” 42.96 (from 1991 to 2009), “vegetables,” 40.61 (from 1990 to 2014), “potentially anticarcinogenic flavonoids,” 37.47 (from 1996 to 2014), and “lipid peroxidation,” 35.22 (from 1991 to 2014), respectively. The sustained strong citation bursts of the top 20 keywords up to 2024 suggested that the field of kaempferol research would continue to attract a significant number of scholars.

VOSviewer was used for “all keywords” analysis. Figure 4C provides a visual representation of the top 30 keywords from global publications on kaempferol after establishing a minimum occurrence threshold of 380 for a keyword to be included. The size of the nodes corresponds to the occurrences; the lines suggest the connections between keywords, and the thickness of the lines indicates the strength of the connection between keywords. The institutes were roughly divided into four clusters based on the degree of occurrences, represented by different colors. Table 6 offers detailed information on the top 30 keywords from the global publications concerning kaempferol. These keywords could be divided into four different directions as follows: (1) keywords related to flavonoids, antioxidant, antioxidant activity, polyphenols, identification, leaves, phenolic-compounds, glycosides, extracts, constituents, acid, phenolic compounds, plants, HPLC, and flavonols; (2) keywords related to kaempferol, quercetin, oxidative stress, expression, inhibition, apoptosis, cells, inflammation, activation, molecular docking, and network pharmacology; (3) keywords related to flavonoid research; and (4) keywords related to *in-vitro* research.

**Table 6:** Top 30 keywords of kaempferol’s global publications.

Rank	Keyword	Occurrence	Rank	Keyword	Occurrence	Rank	Keyword	Occurrence
1	Flavonoids	3,235	11	Phenolic-compounds	764	21	Flavonoid	505
2	Kaempferol	2038	12	Expression	740	22	Cells	468
3	Quercetin	1,608	13	Inhibition	724	23	Inflammation	467
4	Antioxidant	1,363	14	Glycosides	676	24	Phenolic compounds	460
5	Antioxidant activity	1,350	15	Extracts	645	25	Plants	433
6	Polyphenols	939	16	Apoptosis	639	26	Hplc	425
7	Identification	847	17	Constituents	603	27	Activation	413
8	Leaves	814	18	Acid	530	28	Flavonols	398
9	Oxidative stress	777	19	l.	527	29	Molecular docking	398
10	*in-vitro*	771	20	Extract	522	30	Network pharmacology	385

## 4 Discussion

### 4.1 Distribution of kaempferol research

In this study, through the bibliometric and visual analysis of the literature knowledge map in the field of global research related to kaempferol, this study interpreted the research hotspot, research depth, and research frontier in the field of kaempferol, and explored the future development direction of kaempferol. In general, the introduction and application of new scientific and technological methods have enabled numerous renowned research teams to achieve significant advancements in various aspects of kaempferol, including its physical and chemical properties, extraction and preparation processes, and pharmacological mechanisms. In terms of publications, a total 11,214 documents related to the topic of kaempferol were obtained from the Web of Science Core Collection database until February 26, 2024, including articles (*n* = 10,746, 96%) and review articles (*n* = 468, 4%). The global annual number of kaempferol publications was more than 100 documents per year since 2000, and over 500 documents per year since 2018, which further exceeded 1,000 per year since 2022. This indicated that research on kaempferol remained a major focus in the scientific research. Among them, the total number of papers published in China was more than 3,054, accounting for the most documents of the total publication output. Especially since 2014, the number of publications had been rapidly increasing, indicating that research on kaempferol had received increasing attention from the Chinese in the past decade. Among the top 10 most co-cited authors, 4 were from China. However, there was still a problem of insufficient influence globally of China in the research field of kaempferol. As one of the earliest institutions to begin research on kaempferol, the United States of America was another country with more than 947 publications, presenting the highest centrality. Regarding cooperative relationships, frequent collaborations existed between countries/regions and institutions. For instance, Chinese academic institutions represented by Chinese Academy of Sciences, and Egyptian academic institutions represented by National Research Centre NRC, all maintained frequent collaborative relationships with other institutions. In addition, according to this bibliometric study, kaempferol papers mainly contributed to the categories of Food Science Technology, Pharmacology Pharmacy, Biochemistry Molecular Biology, Chemistry Medicinal, Plant Sciences, Chemistry Applied, Chemistry Multidisciplinary, Nutrition Dietetics, Chemistry Analytica, Integrative Complementary Medicine, etc., with at least 600 documents published per category. Our study revealed the hotspots, depth, and frontiers of kaempferol research in the world through bibliometrics and visualization analysis. It pointed out that although China has made significant progress in research quantity and cooperation, its international influence needs to be improved. At the same time, it pointed out that kaempferol research has made in-depth development in many fields such as food, pharmacology, and biochemistry.

### 4.2 Dietary benefits and pharmacological effects of kaempferol

The interest in medicinal plants for their therapeutic potential is expanding worldwide, with a significant portion of their health benefits being attributed to phenolic compounds, notably flavonoids (7). Reports have shown that kaempferol has a broad spectrum of preventative properties, including antioxidant, anticancer, and anti-inflammatory effects, making it beneficial in a daily human diet (50). The inhibition kinetics assay provides evidence that kaempferol can inhibit xanthine oxidase activity reversibly in a competitive manner, which is beneficial to prevent and treat hyperuricemia (51, 52). Besides, some studies have suggested that dietary kaempferol may exert the potential cardiovascular protection effects on human body (53). Kaempferol is also regarded as a dietary anti-inflammatory agent in preventing inflammation risks (54). In addition, studies have shown that supplementation of kaempferol in the diet of obese mice can increase lipid metabolism by down-regulating PPARγ and SREBP1c, reduce lipid accumulation in adipose tissue, and improve antioxidant defense abilities (55). Kaempferol serves as an excellent raw material for the processing of green health foods. Leveraging its bactericidal properties, it can be utilized as a preservative and freshness-retaining agent in the food industry (50). As health awareness increases, nutrition, safety, and health in diet have become key focuses in food development, with functional natural active ingredients gaining global attention. Kaempferol, being abundant in plants and recognized as safe and non-toxic, exhibits a range of biological activities and holds promising potential in the dietary sector.

Kaempferol, a naturally occurring polyphenolic compound, exhibits a wide range of significant pharmacological effects. Regarding the nervous system, kaempferol elicits neuroprotective effects by mitigating inflammation and oxidative stress triggered by microglial cell activation (56, 57). In the digestive realm, kaempferol boasts pronounced anti-inflammatory properties, inhibiting the synthesis and release of diverse inflammatory mediators, thereby diminishing digestive system inflammation and alleviating gastritis, enteritis, and other related symptoms (58, 59). Within the respiratory system, kaempferol showcases antiviral capabilities, inhibiting the proliferation of multiple viruses and contributing to the prevention and treatment of respiratory diseases, including viral colds and viral pneumonia (60, 61). In the circulatory system, kaempferol offers cardiovascular protection by employing mechanisms such as inhibiting apoptosis of vascular smooth muscle cells and modulating blood pressure and lipid profiles (62, 63). For the endocrine system, kaempferol possesses a therapeutic potential in diabetes management, attributed to its ability to regulate insulin secretion and improve insulin sensitivity (64). In the urinary system, kaempferol contributes to the prevention and treatment of urinary disorders by mitigating inflammatory responses and oxidative damage in organs like the kidneys (65). Within the immune system, kaempferol modulates the activity and functionality of immune cells, bolstering the body’s immune defenses and enhancing its resilience against diseases (66, 67). Furthermore, kaempferol demonstrates a broad spectrum of antitumor activity in the reproductive system, combating ovarian cancer, prostate cancer, and other malignancies through mechanisms that include inducing tumor cell apoptosis and inhibiting tumor angiogenesis (68–71). In conclusion, kaempferol’s extensive biological activities underscore its significant pharmacological effects in anti-inflammatory, antioxidant, antiviral, anti-cancer, cardiovascular protection, and neuroprotective protection firmly establishing a theoretical foundation for its utilization in health foods and pharmaceutical products.

### 4.3 Anti-inflammation and antioxidant activity of kaempferol

Recently, natural-sourced drugs are considered the therapeutic strategy for the treatment of inflammatory diseases (8, 72), including rheumatic arthritis, inflammatory bowel disease, periodontitis, etc., which are common diseases seriously harming people’s health. With the progress of modern medical technology, more and more researchers pay attention to the application of kaempferol in the treatment of inflammatory diseases (12). The pharmacological effects of kaempferol in inflammatory diseases are primarily realized through the inhibition of inflammatory factors, anti-oxidative actions, and the prevention of inflammatory cell infiltration (73). The inhibitory effect on inflammatory factors is the key to the anti-inflammatory effect of kaempferol. Besides, kaempferol also exerts anti-inflammatory effects by directly inhibiting the activity of epoxidase and shifting the metabolic pathway of arachidonic acid, thus inhibiting the production of inflammatory response (74, 75). Evidence has demonstrated that kaempferol achieves its anti-inflammatory effects by diminishing the attraction of inflammatory cells and their accumulation during inflammatory responses. It also reduces the expression of inflammation-related mediators such as IL-6, IL-1β, TNF-α, and MIP-2, and exerts inhibitory regulation on pattern recognition receptor Dectin-1 and the pro-inflammatory pathway p38 MAPK (76, 77). Furthermore, another major effect of kaempferol is its antioxidant effect. By directly eliminating the generation of free radicals, kaempferol protects cells from oxidative damage, thus inhibiting the inflammatory response and the further spread of cell damage (19). Generally, reactive oxygen species (ROS) are harmful to cells because their increased level promotes cascade reactions and causes oxidative damage. Kaempferol plays an antioxidant role by aiding the body in clearing the abnormal increased levels of ROS (78). The above researches suggested that kaempferol has the potential in treating inflammatory diseases such as rheumatoid arthritis and inflammatory bowel disease by inhibiting inflammatory factors, antioxidation, and preventing inflammatory cell infiltration.

### 4.4 Antiviral and antimicrobial activity of kaempferol

Currently, viral infection is the most serious health issue that causes an unexpected higher rate of death worldwide (79). The antiviral medicinal plants and the isolated bioactive compounds including kaempferol, apigenin, luteolin, etc., have been considered for further advanced investigations with the aim to develop effective and affordable antiviral drugs (61, 80, 81). Furthermore, kaempferol can also inhibit neuraminidase activities, and has a high inhibitory ability against influenza H1N1 and H9N2 viruses (82, 83). Evidence has demonstrated that kaempferol is effective against DNA viruses such as hepatitis B virus, African swine fever virus, and pseudorabies virus. Besides, kaempferol also performs well in fighting against RNA viruses, namely feline SARS coronavirus, dengue fever virus, etc. (84). Kaempferol is one of the most well-known flavonoids, and it can be found in a variety of herbal and plant families. Kaempferol and its related compounds have antibacterial and antifungal activities in addition to anti-inflammatory effects (85, 86). Meanwhile, kaempferol is also the most effective flavonoid for cell membrane destruction in *E. coli* tested in a study that objectified the findings by showing bacterial proteins leaking into the extracellular environment (87). Kaempferol can significantly inhibit the expression of IL-1β, IL-6, IL-10, TGF-β, TNF-α and other inflammatory factors in the intestinal tract of salmonella-infected chicks, and significantly enhance the expression levels of intestinal mucosal immune factors such as MUC1 and MUC2 (88, 89). Therefore, kaempferol has a certain protective effect on intestinal damage caused by *salmonella enteritidis* infection (90, 91). In addition, oral kaempferol significantly improves the survival rate of mice infected with *klebsiella pneumoniae* (92, 93). Kaempferol may serve as a significant antiviral and antimicrobial agent, holding substantial promise for the prevention and treatment of infectious diseases and thus possessing important research value. As a multifunctional flavonoid, kaempferol has shown great potential in the field of infectious disease prevention and treatment due to its multiple activities such as antiviral, antibacterial, antifungal, and immune regulation. In particular, as an effective and economical therapeutic drug candidate for viral and bacterial infections, it is worthy of further research and development.

### 4.5 Anticancer potential of kaempferol

Evidence has confirmed the anticancer effects of kaempferol in breast cancer, skin cancer, liver cancer, prostate cancer, colon cancer, among others (94). In recent years, an increasing body of evidence has suggested that kaempferol possesses anti-tumor effects through multiple mechanisms, such as interfering with the pericellular phase, inducing the death of the cell through various ways, inhibiting the formation of the tumor-blood vessel, stimulating the immune system of the live host, and acting as the superoxide dismutase in the cell (95, 96). Therefore, kaempferol holds a promising clinical application prospect in the prevention and treatment of tumors. Studies have also shown that kaempferol can inhibit the proliferation and induce apoptosis of breast cancer SK-BR-3 cells *in vitro*, which may be related to the regulation of Notch1 and Cleaved caspase-3 protein expression (96–99). Meanwhile, kaempferol can inhibit the proliferation of SGC-7901 cells and induce cell apoptosis by the apoptosis mechanisms of increasing the release level of intracellular ROS (100–102). Besides, kaempferol also performs well in inhibiting the maturation of dendritic cells induced by peripheral blood mononuclear cells in gastric cancer patients (103, 104). Furthermore, kaempferol may play an anti-colon cancer role by regulating the expression of MMP1, MMP2, and MMP9 protein, thereby increasing the expression level of miR-339-5p to mediate PKM alternative splicing reversing aerobic glycolysis in colon cancer cells (105–109). Moreover, kaempferol markedly suppresses HepG2 cell proliferation and combats liver cancer by increasing BAX and JUN protein expression and decreasing CDK1 protein levels (110, 111). In addition, kaempferol plays an anti-prostate role by inducing cycle arrest of prostate cancer cells, inducing apoptosis of prostate cancer cells, and inhibiting the proliferation of prostate cancer cells (71, 98). Briefly, kaempferol exhibits significant inhibitory effects on various cancers through multiple anticancer mechanisms, including interfering with the cell cycle, inducing apoptosis, inhibiting tumor angiogenesis, activating the immune system, and regulating gene expression. This suggests a broad clinical application prospect in cancer prevention and treatment, but more clinical studies are needed to confirm its efficacy.

### 4.6 Cardiovascular protection of kaempferol

Heart failure is a complex clinical syndrome characterized by the heart’s inability to pump enough blood into the body through structural and/or functional cardiac abnormalities (112). Studies have suggested that kaempferol has effects of anti-cardiomyocyte hypertrophy and interstitial fibrosis (113, 114). Kaempferol can reduce LPS-induced apoptosis of H9c2 cells in rat myocardium, possibly through the TLR4/NF-κB pathway (115). Cardiomyocytes injured by local anoxia/reoxygenation (A/R) can be treated with kaempferol. It has been observed that kaempferol can significantly enhance the expression of Bcl-2 via the SIRT1-mediated mitochondrial pathway, thereby exerting protective effects against apoptosis in cardiomyocytes induced by A/R injury (116, 117). Kaempferol can reduce the activity of CK, LDH and MDA, and increase the activity of SOD to protect cardiomyocytes from hypoxic injury (25, 113, 118). Research has indicated that by regulating the PI3K/AKT signaling pathway, kaempferol may attenuate atherosclerosis (119). Besides, kaempferol can significantly reduce blood pressure, inhibit Scr and BUN levels, improve renal function, alleviate renal fibrosis damage, inhibit intrarenal inflammation and oxidative stress, and possibly prevent hypertension and its target organ damage by inhibiting the TGF-β1/β-arrestin1 pathway (120). Kaempferol has also been found to have a protective effect on cardiovascular diseases through mechanisms such as inhibition of TNF-α production and activation of NF-κB; activation of Ca2^+^ − activated K^+^ channels; enhancement of NOS activity of endothelial cells by stimulating arterial dilation; and mitigation of oxidative stress (121, 122). Heart failure is a complex clinical syndrome, the core of which is the insufficient blood pumping capacity of the heart. Kaempferol exhibits significant protective effects on heart failure and related cardiovascular pathological processes through multiple pathways, such as anti-myocardial cell hypertrophy, fibrosis, and apoptosis. These findings not only reveal the extensive role of kaempferol in cardiovascular protection, but also provide scientific evidence for its potential as a low-risk treatment method, emphasizing its great prospects in clinical application. Despite the need for further clinical research to confirm kaempferol’s role in cardiovascular protection, it is still considered a potentially low-risk treatment and highlights the substance’s promising potential for clinical use.

### 4.7 Neuroprotective protection of kaempferol

Numerous studies have indicated that kaempferol possesses therapeutic effects for neurological diseases such as Alzheimer’s disease, Parkinson disease, major depressive disorder, anxiety disorders, neuropathic pain, etc. (123, 124). Besides, previous research has demonstrated kaempferol as a neuroprotective protection agent for secondary spinal cord injury by reducing oxidative stress and inflammatory response through the down-regulation of ROS dependent MAPKs- NF-κB and the pyroptosis signaling pathway (125). Meanwhile, studies have also suggested that kaempferol has the neuroprotective effect in reducing neuropathic pain by regulating the TLR4/NF-kappa B signaling pathway (126). In the oxygen–glucose deprivation/reperfusion (OGD/R)-induced neuronal injury mouse model, kaempferol provides neuronal protection from OGD/R-induced ferroptosis partly by activating the Nrf2/SLC7A11/GPX4 signaling pathway (127). Moreover, kaempferol has also been found to be a potent mitophagy modulator in stimulating neuronal health and brain homeostasis (128). Furthermore, kaempferol can protect the nervous system damage caused by oxidative stress by inhibiting oxidative stress. For example, kaempferol has been shown to significantly protect SH-SY5Y neuronal cells, mitigating damage to major neuron cells by reducing protease cleavage and nuclear apoptosis. Additionally, it significantly lowers levels of ROS and mitochondrial hydroxyl radicals (129–131). Collectively, evidence supports that kaempferol offers neuroprotective capabilities, positioning it as a candidate for development into a neuroprotective therapeutic agent. In summary, kaempferol exhibits significant therapeutic effects on various neurological diseases by reducing oxidative stress, regulating signaling pathways, and promoting mitochondrial health through multiple mechanisms, strengthening its scientific basis as a candidate for neuroprotective therapeutic agents.

### 4.8 Other biological activities of kaempferol

Kaempferol is also used to treat numerous acute or chronic diseases, including intervertebral disc degeneration, colitis, post-menopausal bone loss, acute lung injury, liver injury, obesity, diabetes, hypertrophic scar, among others (132, 133). Research has demonstrated that kaempferol may interact with some amino acid residues within the active site of alpha-glucosidase, thereby achieving the purpose of treating diabetes (134). Furthermore, kaempferol can improve insulin resistance in db/db mice by inhibiting NLPR3 inflammatory-mediated adipose tissue inflammation (135). In addition, as network pharmacology and molecular docking technologies develop and are applied, an increasing number of mechanisms by which Traditional Chinese Medicine (TCM) compounds treat diseases are being uncovered. Kaempferol is frequently identified as a key component in the material basis of these TCM formulae (136). A study explored the therapeutic effect of an anticancer traditional Chinese medicine (TCM) formula containing kaempferol on non-small cell lung cancer (NSCLC) based on the methodology of network pharmacology (137). Researchers first predicted the potential targets of kaempferol through network pharmacology databases and then constructed a compound-target-disease network by integrating relevant targets of NSCLC. Subsequently, the impact of kaempferol and its formula on the proliferation, migration, invasion, and apoptosis of NSCLC cells was verified through *in vitro* cellular experiments. Furthermore, scholars utilized network pharmacology to predict the potential targets of a kaempferol-containing TCM formula for the prevention and treatment of high-altitude pulmonary edema (HAPE), and the preventive and therapeutic effects were validated through animal experiments (138). Therefore, Through the network pharmacology method, researchers can systematically analyze the pharmacological mechanism of kaempferol in Chinese medicine compounds, and provide scientific basis for its optimization and expansion in clinical application. Evidence has also indicated that kaempferol can improve the oxidative and inflammatory damage of knee osteoarthritis rat chondrocytes by inhibiting the ROS/TXNIP pathway, thereby regulating the levels of oxidative markers and inflammatory factors (139). Furthermore, kaempferol can also increase the vitality and proliferation of rat preosteoclasts, and promote osteogenesis through the Wnt/β-catenin signaling pathway, which may be the mechanism of osteoporosis prevention and treatment (140, 141). Briefly, kaempferol not only shows potential in the treatment of various acute and chronic diseases, but also affects various physiological and pathological processes through complex molecular mechanisms. Its multi-pathway and multi-target characteristics play an important role in traditional Chinese medicine compound and modern pharmacological research, indicating a broad prospect for further exploration of kaempferol and its related applications in the future.

## 5 Conclusion

Kaempferol, a flavonoid compound, is widely present in natural plants such as fruits, vegetables, and traditional Chinese medicinal herbs, exhibiting diverse biological activities. Herein, information visualization techniques were employed to elucidate the research progress, hot topics, and frontiers in kaempferol. While the annual publication output in China has far exceeded that in the United States of America in recent years, its academic influence is still left far behind. In addition, scholars, institutions, and representative literature playing important roles in this field were hereby identified. Keyword analysis demonstrated the anti-inflammatory and antioxidant activity of kaempferol and its potential dietary benefits as the main research direction, with network pharmacology and molecular docking identified as the latest hot technical means. Given its prominent anti-inflammatory and antioxidant properties, research on kaempferol’s bioactivity has progressively uncovered and expanded into areas such as anti-viral capabilities (against influenza, novel coronavirus), cardiovascular protection (preventing myocardial ischemic injury, atherosclerosis, and hypertension, etc.), neuroprotection (against neuritis, Alzheimer’s disease, myelitis, etc.), and anti-diabetic effects. Kaempferol significantly reduces the risk of cancer, particularly in pancreatic cancer and colorectal adenoma, indicating its potential application value in cancer prevention and treatment, and providing crucial clues for the future development of novel anticancer drugs. Furthermore, its anti-inflammatory and antibacterial properties make kaempferol a promising candidate for the treatment of inflammatory and infectious diseases, potentially helping to mitigate overuse of antibiotics and issues related to antibiotic resistance. Kaempferol’s antioxidant and antiviral characteristics aid in protecting cells from oxidative damage and viral infections, thereby playing a significant role in the prevention and treatment of related diseases. Additionally, kaempferol regulates blood sugar and lipid levels, contributing to the prevention and treatment of metabolic diseases like diabetes and atherosclerosis. These discoveries offer new perspectives for the development of novel hypoglycemic and hypolipidemic drugs, thus improving the global prevention and control of metabolic diseases. Consequently, future research directions for kaempferol can be explored from the following aspects: (1) Further elucidating the specific mechanisms of kaempferol in different types of cancer, particularly its synergistic effects with other anticancer drugs, to provide a scientific basis for the development of novel anticancer drugs. (2) Investigating the potential applications of kaempferol in other fields such as inflammatory diseases, infectious diseases, and metabolic disorders, thereby expanding its clinical application scope. (3) Conducting a comprehensive toxicity assessment of kaempferol to ensure its safety in clinical use. Simultaneously, studying its metabolic differences and pharmacokinetic characteristics across different populations will provide a basis for individualized medication. To this end, the dietary advantages and multiple pharmacological effects of kaempferol underscore its clinical significance and highlight its crucial role in disease prevention and treatment.

## 6 Limitation

Despite the findings, the present bibliometric analysis only included publications in the Web of Science Core Collection (WoSCC) database, overlooking other databases such as PubMed, Cochrane library, and Google Scholar. However, it should be noted that WoSCC is widely recognized as one of the most authoritative scientific literature search platforms, covering the vast majority of research on kaempferol and maintaining a certain degree of representativeness.

## Data Availability

The original contributions presented in the study are included in the article/supplementary material, further inquiries can be directed to the corresponding author.

## Footnotes

^1^https://www.ncbi.nlm.nih.gov/mesh
